# Elucidating the evolving role of cuproptosis in breast cancer progression

**DOI:** 10.7150/ijbs.98806

**Published:** 2024-09-09

**Authors:** Zhanyong Zhu, Keyu Zhu, Jun Zhang, Yunhua Zhou, Qi Zhang

**Affiliations:** 1 Department of Plastic Surgery, Renmin Hospital of Wuhan University, No. 238 Jiefang Road, Wuhan, 430060, Hubei Province, China.; 2 Department of Plastic and Cosmetic Surgery, Tongji Hospital, Tongji Medical College, Huazhong University of Science and Technology, Wuhan, 430030, Hubei, China.; 3 Department of Thyroid and Breast Surgery, Shenzhen Qianhai Shekou Free Trade Zone Hospital, Shenzhen, 518067, China.; 4 Department of Wound Repair Surgery, Liyuan Hospital, Tongji Medical College, Huazhong University of Science and Technology, Wuhan, 430062, Hubei Province, China.; 5 Xianning Medical College, Hubei University of Science & Technology, Xianning, 437000, Hubei, China.

**Keywords:** breast cancer, cuproptosis, copper, cuproptosis-related genes, diagnosis, therapy

## Abstract

Breast cancer (BC) persists as a highly prevalent malignancy in females, characterized by diverse molecular signatures and necessitating personalized therapeutic approaches. The equilibrium of copper within the organism is meticulously maintained through regulated absorption, distribution, and elimination, underpinning not only cellular equilibrium but also various essential biological functions. The process of cuproptosis is initiated by copper's interaction with lipoylases within the tricarboxylic acid (TCA) cycle, which triggers the conglomeration of lipoylated proteins and diminishes the integrity of Fe-S clusters, culminating in cell demise through proteotoxic stress. In BC, aberrations in cuproptosis are prominent and represent a crucial molecular incident that contributes to the disease progression. It influences BC cell metabolism and affects critical traits such as proliferation, invasiveness, and resistance to chemotherapy. Therapeutic strategies that target cuproptosis have shown promising antitumor efficacy. Moreover, a plethora of cuproptosis-centric genes, including cuproptosis-related genes (CRGs), CRG-associated non-coding RNAs (ncRNAs), and cuproptosis-associated regulators, have been identified, offering potential for the development of risk assessment models or diagnostic signatures. In this review, we provide a comprehensive exposition of the fundamental principles of cuproptosis, its influence on the malignant phenotypes of BC, the prognostic implications of cuproptosis-based markers, and the substantial prospects of exploiting cuproptosis for BC therapy, thereby laying a theoretical foundation for targeted interventions in this domain.

## Introduction

Breast cancer (BC) remains the most prevalent and high-mortality type of female tumor worldwide, imposing a heavy social burden 1. With the intensive development of diagnostic and therapeutic technologies, individualized treatment strategies based on molecular typing have led to a significant improvement in the prognosis of BC patients 2,3. However, the prognosis of some high-risk BC types or advanced BC is still poor 4. The genetics, pathology, and molecular biology of patients are profoundly altered, and continued exploration will bring about profound transformations in the management of BC 5.

Cell death, as one of the metabolic processes, is of importance for the maintenance of biological life, along with cell proliferation and differentiation 6,7. Different programmed cell deaths (PCDs) are extensively involved in tumor evolution, including apoptosis, necroptosis, pyroptosis, ferroptosis, and cuproptosis 8. PCDs are cellular suicide mechanisms that have arisen in living organisms during a long evolutionary process and are closely related to a variety of pathophysiological phenomena, including cell fate, immune regulation, tumors and infectious diseases, among others 9. Notably, PCD involves specific intracellular gene regulatory molecular programs, and many neoplastic diseases are associated with defects in one or several of the PCD processes 10. In particular, copper homeostasis in the body is sustained by modulating copper absorption, transport, and excretion 11. Copper homeostasis is fundamental to cellular homeostasis since the accumulation of intracellular copper induces oxidative stress and disrupts many cellular functions 12.

Cuproptosis begins with the binding of copper to lipolysis in the tricarboxylic acid (TCA) cycle, contributing to consequential protein aggregation, proteotoxic stress, and eventually cell death 13. Cuproptosis is dysregulated in BC tissues, and cancer cells have a higher copper requirement compared to undivided cells. High heterogeneity in the expression variation of cuproptosis-related genes (CRGs) in BC reveals that the imbalance of CRG expression plays a pivotal role in BC development. Abnormal copper accumulation promotes the malignant transformation of cells 14. CRGs and BC progression are inextricably linked 15. For example, ATP7B and dihydrolipoamide S-acetyltransferase (DLAT) protein expression showed a high expression pattern in BC samples and had an impact on the tumor cell cycle. The novel constructed risk signatures of CRGs have predictive properties for the prognosis, tumor microenvironment (TME), and immunotherapy of BR patients 16. Some CRGs, DLAT, solute carrier family 31 member 1 (SLC31A1), ATP7A, and ATP7B, are distinctively associated with overall survival (OS) in BC patients 17. Zou *et al.* established a 12-gene cell death index (CDI), and patients in the triple negative breast cancer (TNBC) cohort with a high index had worse prognostic outcomes 18. The CDI score correlated with ICs and sensitivity to standard adjuvant chemotherapy and palbociclib.

Given the importance of cuproptosis and its involvement in multiple aspects of BC, we systematically review in this article the basic concepts of cuproptosis, the impact of cuproptosis on the malignant biological behaviors of BC, the prognostic value of cuproptosis based on cuproptosis, as well as the huge potential of cuproptosis for BC treatment. An in-depth understanding of cuproptosis and its underlying molecular mechanisms is beneficial for appreciating the evolution of BC and screening cuproptosis-targeted therapeutic strategies.

## Copper metabolism and cuproptosis *in vivo*

Copper, an essential trace element in the animal body, is primarily found in muscle, bone, and liver 19. Owing to its redox properties, copper can serve as a cofactor for key enzymes involved in a variety of biological processes, including energy conversion, antioxidant, intracellular oxidative metabolism, and epigenetic modifications 20.

The main source of copper in the body is absorbed from food through the duodenum and small intestine 21. The six-transmembrane epithelial antigen of the prostate (STEAP) can reduce Cu2+ from food to Cu+, which then enters the cells through the mediation of copper transport protein 1 (CTR1) 22. Cu2+ can also be transported into the cells via divalent metal transporter 1 (DMT1), but it cannot be utilized directly 23. After being absorbed by the epithelial cells, Cu can be transported to the other side of the epithelium via the copper molecular chaperone antioxidant 1 (ATOX1), then secreted into the bloodstream through the ATPase copper transporting alpha (ATP7A) 24. Soluble carriers in the blood that bind to copper ions, such as albumin, copper-green proteins, and histidine, can bind to copper and then transport it to the liver, which is the primary organ for storing and excreting copper 25,26. Copper storage proteins, such as metallothionein and glutathione, can mediate Cu storage by chelating with Cu+, and excess Cu is secreted into the bile, mainly through the ATPase copper transporting beta (ATP7B) on hepatocytes, and then gets excreted out of the body 27,28.

Collectively, these processes mediate copper homeostasis at the macroscopic level *in vivo*. At the cellular level, copper enzymes, copper molecular chaperones, and membrane transport proteins, synergistically regulate the uptake, efflux, and utilization of copper, thus maintaining copper homeostasis 29,30. Copper transporter 1 (CTR1) and STEAP are cooperatively responsible for transporting copper into the cells, while copper molecular chaperones, such as CCS, Atox 1, and Cox17, are in charge of intracellular copper transport 31,32. CCS delivers copper to superoxide dismutase 1 (SOD1), which is involved in reactive oxygen species (ROS) scavenging and maintenance of copper homeostasis 33. ATOX1 binds Cu+ and transfers it to ATP7A and ATP7B in the trans-Golgi network, enhancing the synthesis of cuproenzymes 34. When intracellular copper levels are overly high, ATP7A and ATP7B can facilitate the efflux of excess copper through translocation to the cell membrane 35. Cox17 can target Cu+ to the mitochondrial membrane gap and insert it into cytochrome oxidase C (CCO) via COX11 or SCO1, participating in cellular metabolism 36,37. Abnormalities in these processes lead to disturbed copper metabolism and disrupted copper homeostasis.

Maintaining normal copper homeostasis is necessary for normal life activities. Lack of copper ions may lead to deficiencies in the activity of multiple enzymes and the development of disease 38. However, excessive copper accumulation can also harm cells. Copper ions undergo redox reactions accompanied by electron transfer and ROS production, capable of damaging a variety of cellular biomolecules 39,40. Elevated copper levels are also linked with the severity and progression of several cancers. Copper can promote tumorigenesis and progression by enhancing cell proliferation, inducing drug resistance in tumor cells, stimulating angiogenesis, and promoting tumor cell migration 41,42. On the other hand, the application of copper alone or together with ion carriers can induce cancer cell death, such as cuproptosis 43,44.

**Figure 1:**
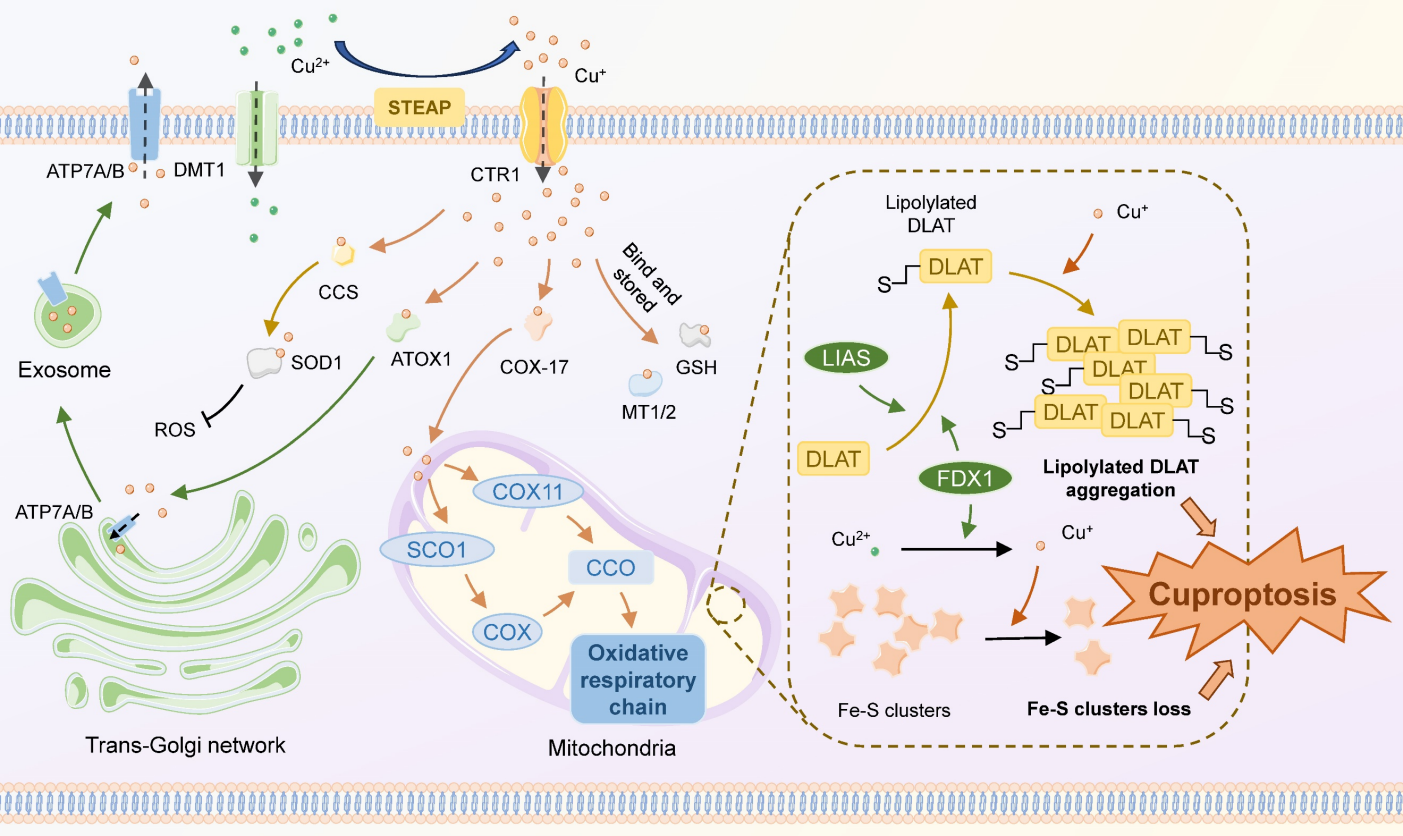
** Mechanisms of cuproptosis.** Extracellular copper exists primarily in the form of Cu2+, which can directly enter the cell via DMT1 or travel through CTR1 after being reduced to Cu+ by STEAP. Cu+ enters the cell and binds to different copper ion carriers, thus avoiding its cytotoxicity, and there are four main destinations: 1) Binds to GSH or MT1/2 to be stored, forming an unstable copper pool; 2) Binds to CCS, then translocates to and inserts into SOD1, which participates in the detoxification of ROS; 3) Binds to ATOX1 and is targeted for translocation to ATP7A/B in the trans-Golgi network, contributing to copper enzyme synthesis or copper efflux; 4) Binds to COX17 and targets for transporting to the mitochondrial membrane interstitials, inserting into CCO and engaging in the cytosolic oxidative respiratory chain. The classical mechanism of cuproptosis primarily involves the aggregation of lipoylated TCA cycle proteins and the loss of Fe-S clusters in the mitochondria. FDX1 and LIAS regulate the lipoylation of mitochondrial proteins. High intracellular concentrations of Cu+ can bind to lipoylated proteins and foster their aggregation. Cu+ can also destabilize the Fe-S Cluster, leading to a reduction in the Fe-S Cluster. These can lead to Cu-induced proteotoxic stress and ultimately cell death.

Cuproptosis is a regulatory cell death pattern distinct from other oxidative stress-related cell death patterns 45. The morphological features of cuproptosis are similar to those of apoptosis, characterized by mitochondrial crumpling, cell membrane rupture, endoplasmic reticulum damage, and chromosome breaks 46. In this process, Cu+ directly binds to lipoylated mitochondrial protein fractions of the tricarboxylic acid (TCA) cycle and acts on Fe-S clusters, inducing the aggregation of lipoylated proteins and the reduction of Fe-S clusters, ultimately resulting in cellular death mediated by proteotoxic stress 47 **(Figure 1)**. Some critical components play important roles in this process. For example, as a key gene in cuproptosis, FDX 1 is involved in proteolipid acylation and reduction of Cu2+, but its exact mechanism still requires further interpretation. Several other CRGs, including those encoding LIPT 1, LIAS, DLD, DLAT, PDHA 1, and PDHB, are also significantly involved in this procedure.

## The roles and mechanisms of cuproptosis in remodelling BC progression

As an oncogene, cyclin-dependent kinase inhibitor 2A (CDKN2A) can encode p16 and p14, often expressed as homozygous deletions in a variety of cancers, such as oral squamous cell carcinoma and melanoma 48,49. Deletions, point mutations, promoter hypermethylation, and transcriptional abnormalities of the CDKN2A gene, are responsible for the inactivation of the CDKN2A gene, resulting in cell cycle dysregulation. CDKN2A is identified as a key CRG whose high expression was predictive of poor outcomes through model screening constructed by artificial intelligence 50. Knockdown of CDKN2A using siRNA technology resulted in a significant decrease in Zeb1, vimentin, and MMP9, indicators related to BC cell migration and metastasis, as well as down-regulation of the expression of MEGEA4, phosphorylated STAT3, PD-L1, and caspase-3. Hence, CDKN2A/MAGEA4 was a pathway that was critically associated with BC chemosensitivity, invasion, and metastasis. In another similar bioinformatic study, CDKN2A was also recognized as a pivotal modulator influencing TNBC course by driving ferroptosis and cuproptosis 51. Histochemical results of multiple samples showed that c-Myc was strongly associated with BC stemness and significantly correlated with cuproptosis-induced cell death 52. Cuproptosis is an underlying downstream of c-Myc-mediated malignant changes in tumors. DLAT fulfils a similar role in BC. Sha *et al.* found that high level of DLAT was associated with resistance to HER2-targeted therapy, and BC sensitivity to trastuzumab was enhanced after knocking down DLAT 53. Dihydrolipoyl dehydrogenase (DLD) is another critical CRG, but its role in BC prognosis remains unclear. Xu *et al.* investigated the potential impact of DLD on BC progression 54. Knockdown of DLD in BC cell lines MDA-MB-468 and SK-BR-3, significantly inhibited cell migration, invasion, and proliferation. High DLD expression in tumor regions was associated with PD-L1 and macrophages, while stromal regions showed an increase in CD4+ T cells and macrophages.

Alternatively, some metabolites may affect the progression of BC by interfacing with CRGs. For example, butyrate, a common short-chain fatty acid (SCFA), has been shown to regulate the development of BC, though its underlying mechanisms were previously unclear. Zhang *et al.* found that SCFA levels were lower in fecal samples from BC patients compared to controls 55. Butyrate significantly inhibited the viability, migration, and invasion of T47D cells in a dose-dependent manner and effectively suppressed tumor growth in animal models. Mechanistically, butyrate inhibited the expression of Toll-like receptor 4 (TLR4), and promoted the expression of CRGs PDXK and SLC25A28. Overexpression of TLR4 reversed the effects of butyrate on PDXK and SLC25A28 expression and on the malignant behavior of T47D cells. These findings suggest that CRGs are important potential prognostic markers in BC, offering new therapeutic targets and avenues for BC treatment.

**Figure 2:**
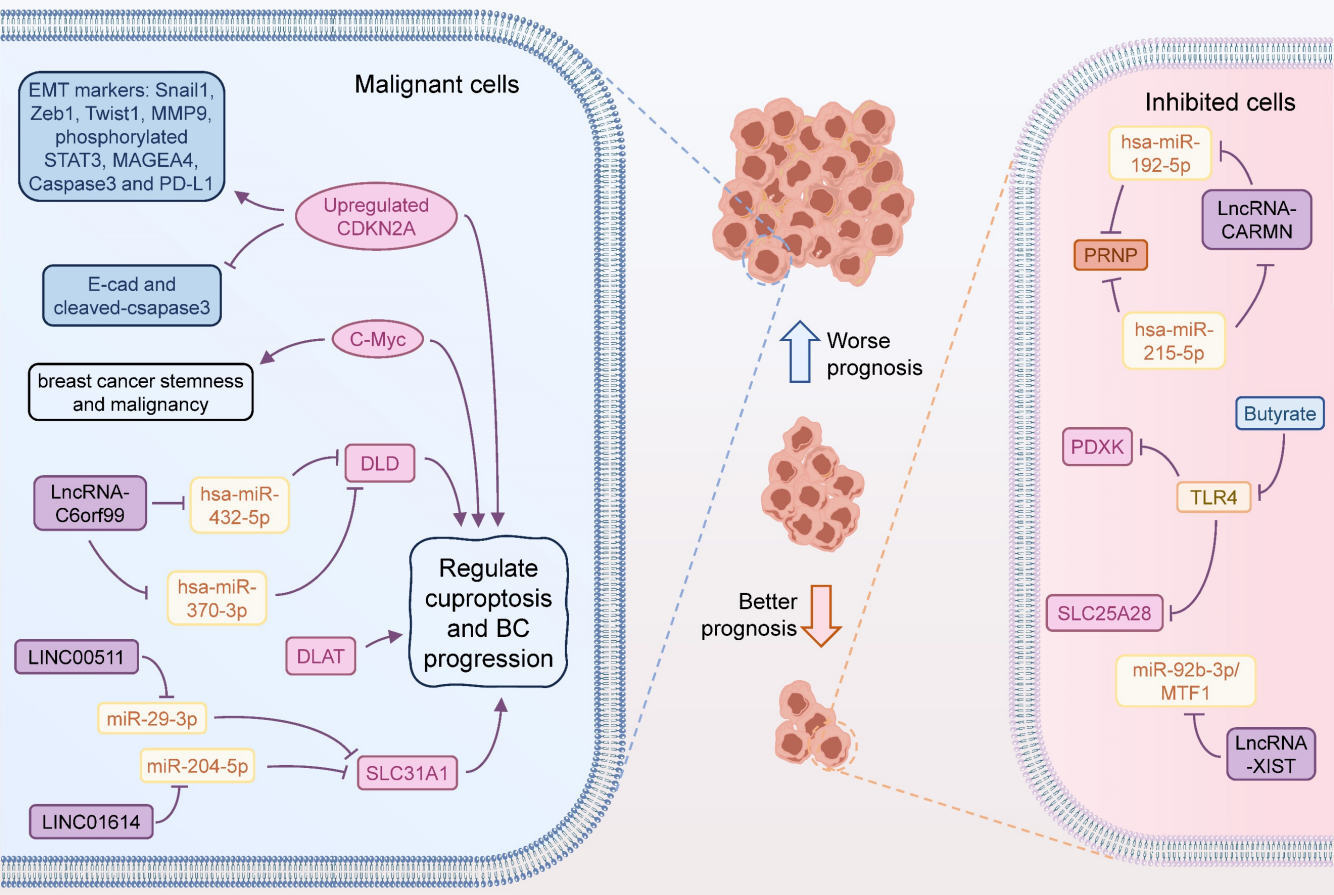
** Cuproptosis-related functions in BC progression.** Some CRGs are involved in the development and progression of BC. Inhibition of CDKN2A downregulates the expression of a series of proteins associated with tumor proliferation and migration, leading to a better prognosis of BC. LncRNA-C6orf99 can enhance the expression of DLD by regulating the expression of hsa-miR-370-3p and hsa-miR-432-5p, thus strengthening the tolerance of BC cells to endocrine therapy. LINC00511 and LINC01614 could act on miR-29-3p and miR-204-5p, respectively, to enhance the expression of SLC31A1, which was associated with worse prognosis of BC. Increased c-Myc expression may also promote BC progression through cuproptosis. LncRNA-XIST can inhibit BC progression by targeting the miR-92b-3p/MTF1 axis. LncRNA-CARMN can promote PRNP expression by regulating hsa-miR-192-5p and hsa-miR-215-5p, thereby inhibiting the development of BC. Butyrate promotes the expression of PDXK and SLC25A28 by inhibiting TLR4, thereby promoting cuproptosis in tumour cells.

CeRNA is a complex regulatory network between ncRNAs and coding RNAs in the cell via miRNAs 56,57. The core principle of ceRNA network is to form a competitive relationship between mRNAs, lncRNAs, circRNAs, through the competitive binding mechanism of miRNAs 58,59. This interaction of multiple makes the intracellular regulatory network more flexible and helpful in adapting to different biological environments 60. By using bioinformatic analysis of multiple databases, Lian *et al.* revealed that SLC31A1 was abundantly expressed in BC tissues and cell lines and was intimately connected to both recurrence-free survival (RFS) and distant metastasis-free survival (DMFS) 61. Functionally, SLC31A1, which was involved in copper ion transport, was found to be associated with m6A-related genes, especially YTHDF3. In-depth, the up-regulation of SLC31A1 expression mediated by the LINC00511-miR-29-3p axis was implicated in the modulation of copper ion transport and poor prognosis of BC, and was positively linked to the tumor immune cell infiltration, immune markers, and cancer-associated fibroblasts (CAFs) 61. Similarly, lncRNA XIST was also found to regulate the miR-92b-3p/MTF1 axis for shaping BC progression, deserving further exploration by *in vitro* and *in vivo* assays 62. In another multi-method bioinformatic study, LINC01614 likewise facilitated the expression of SLC31A1 by inhibiting miR-204-5p and was involved in BC progression. Thus, LINC01614/miR-204-5p/SLC31A1 is also a regulatory chain related to cuproptosis and was critical for BC 63. In addition, PRNP was identified as a core CRG involved in tumor progression in BC. hsa-miR-192-5p and hsa-miR-215-5p were confirmed to target regulatory PRNP by PPI and immunoassay, and a ceRNA regulatory network was identified, including mRNAPRNP/miRNA hsa-miR-215-5p and hsa-miR-192-5p/lncRNA CARMN axis 64. Zhang *et al.* constructed a risk model for Estrogen receptor-positive (ER+) BC consisting of 4 CRGs, including DLD, DBT, DLAT, and ATP7A, that predicted immune infiltration, immune function, ICs, characteristic gene changes, and pathway activation in different scoring risk subgroups 65. In this system, the authors identified two ceRNA action networks lnc RNA C6orf99/hsa-miR-370-3p and hsa-miR-432-5p/DLD, and that DLD was a core target associated with resistance to ET through cuproptosis **(Figure 2).**

## Cuproptosis-related applications in BC diagnosis

Mammography imaging and magnetic resonance imaging, histopathologic testing, and serologic screening methods, have been common clinical screening tools for BC in the present 66,67. Several novel techniques, including the applications of microwave imaging and artificial intelligence, offer new possibilities for the early diagnosis and treatment of BC 68,69. However, postoperative failure, metastasis, relapse, and chemoresistance, are the primary factors contributing to therapeutic failure in BC patients, especially TNBC. Currently, desirable predictive models for these factors are still lacking 17,70,71. Cuproptosis regulates a wide range of biological functions and possesses a contributory function in shaping the complexity and diversity of the TME, then its associated hallmarks are potentially the basic unit of BC prediction 72,73. Models based on multiple CRGs and signatures are capable of responding to a variety of clinical prognostic evaluations, tumor mutation burden (TMB), immune cell infiltration and activity, immune checkpoint, and chemotherapeutic drug sensitivity 74-76. Here, we divide this section into single CRG-based, multiple CRGs, and combined CRG-related genes.

### Models based on single CRG

SLC31A1 is a critically endorsed class of copper transporters that impacts copper uptake at the cell membrane by functioning as a homotrimer, and its overexpression promotes greater copper uptake 77. As an influential player in the cuproptosis gene family, SLC31A1 has been confirmed to be highly correlated with poor prognosis in a range of tumor types, including BC, cervical cancers, head and neck squamous cell carcinoma, and esophageal carcinoma (ESCA) 63,78. Li *et al.* integrated SLC31A1 with other clinical parameters, such as age, T-stage, N-stage, and clinical staging to construct a predictive model, demonstrating favorable predictive effectiveness for both near-term and long-term OS 17. High expression of SLC31A1 was statistically associated with immunity, metabolic dysregulation, and treatment responsiveness to paclitaxel and CTLA4.

Pyruvate dehydrogenase E1 component subunit alpha (PDHA1) is a key gene involved in cuproptosis and consequently in the glucose metabolism reprogram of cancer cells 79. PDHA1 is implicated in a variety of signaling pathways in the malignant evolution of tumors, including DNA damage, cell invasion, immunosuppression, and angiogenesis 80. PDHA1 is potentially a prognostic and immune-related parameter for multiple cancers. For example, Huang *et al.* demonstrated through multiple validation and characterization that PDHA1 was a regulator of BC malignant progression and that PDHA1 expression was tightly correlated with the infiltration of a variety of immune cells, including CD4+ T cells, macrophage subsets, and mast cells 81. Thus, this study demonstrated that PDHA1 was an independent factor in BC prediction and was expected to be a novel immunotherapeutic target.

### Models based on multiple CRGs

Models based on multiple differentially expressed CRGs are able to capture more BC patient information than a single CRG and may be more efficient in terms of accuracy 82,83. After discovering the pivotal role of DLAT in BC, Sha *et al.* constructed a risk score model for HER 2+ BC patients based on CRGs, and confirmed DLAT as an independent prognostic factor for HER2+ BC patients in further analysis 53. Xia *et al.* matched 21 known CRGs with differentially expressed ncRNAs in BC, subsequently screening them with machine learning algorithm 84. They ultimately chose five key CRGs from these to construct a diagnostic model together, which was able to predict the molecular subtypes of BC accurately, providing a new perspective on personalized treatment for BC patients. Zhu *et al.* constructed 6 CRGs-comprising risk signatures for BC prediction 85. The model demonstrated excellent capabilities to delineate high-risk and low-risk groups, to evaluate higher than TNM staging, and to assess immune responsiveness. Zheng *et al.* reported a signature based on CRGs, and emphasized that patients in the high-risk group classified according to the model had lower OS, and there were significant differences between the high- and low-risk groups in terms of OS, immune status, and sensitivity to chemotherapy 86. Jiang *et al.* successfully built a risk model based on 6 CRGs, comprising DKN2A, MTF1, PDHA1, DLD, LIPT1, and FDX1. The model line was predictive of pre-pTNM, MSI, drug sensitivity, and immune infiltration characteristics in BC patients 62.

ER+ BC is the most common subtype of BC and is accompanied by features such as immune stress and epigenetic modifications 87. The treatment of ER+ BC consists of interventions that inhibit estrogen secretion and/or directly target the ER, improving patient outcomes 88. However, the resistance and immune profile of such therapies require clearer predictive modalities and indications. Fan *et al.* constructed a risk scoring system by using CRGs for ER+ early BC, showing that High expression of FDX1, LIAS, LIPT1, DLD, PDHB, and ATP7B and low expression of CDKN2A were related to better RFS 89. This model specified a higher risk of recurrence with a high CRG score and therefore could be used synergistically as a complement to conventional clinical parameters for long-term prognostic evaluation of ER+ early BC.

### Models based on combined CRG-related genes

Models constructed based on multiple composite genes are also common approaches nowadays, including those based on PCD-associated ncRNAs, PCD-associated mRNAs, or Mendelian randomization 90,91. In BC, cuproptosis-related lncRNAs (CRLs) are the most common type of ncRNAs used to construct BC prediction models. In contrast, cuproptosis-related miRNAs and circRNAs based on cuproptosis have barely been mentioned.

CRLs are instrumental in mediating the biological functions of BC with the potential to predict BC prognosis and sensitivity to various therapies 92. The resistance biomarker expressions of CDK4/6 (CCNE1, E2F1, and E2F2) and PARP (BRCA1/BRCA2) were lower in low-risk patients, suggesting that low-risk patients were more prone to the application of these two inhibitors. Utilizing the TCGA database in combination with machine learning, Li *et al.* constructed a cuproptosis-associated lncRNA-based prediction model, which exhibited excellent performance in predicting the prognosis, tumor mutation burden, and responsiveness to immunotherapy in BC patients 93. Similarly, Sun *et al.* also screened differentially expressed cuproptosis-associated lncRNAs in BC samples 94. Cellular experiments confirmed that AC104211.1 and LINC01863 influenced BC cell proliferation, while the screened drugs, Trametinib, 5-fluorouracil, and AICAR significantly inhibited the viability of MCF-7 cells. Xu *et al.* found the cuproptosis genes FDX1, PDHA1, and DLAT were remarkably down-regulated in BC tissues, furthermore, they identified a unique model BCCuS based on cuproptosis-related 2-lncRNAs USP2-AS1 and NIFK-AS1 for BC prediction 95. The BCCuS model had high prognostic predictive performance and was strongly associated with high TMB and immune assessment.

Li *et al.* also constructed a characteristic predictive model using 5 CRLs, C2orf91 (LINC02898), PRKAR1B-AS1, AC012213.3, AL137847.1, MFF-DT, and the pathways of these CRLs were closely related to immune responsiveness 96. The model was able to respond well to the high sensitivity of multiple drugs to high risk, including Lapatinib, Sunitinib, Phenformin, and Idelalisib. Li *et al.* used 10 CRLs for constructing a risk signature for BC risk stratification, which obtained desirable predictive capabilities 97. Yu *et al.* constructed a risk model based on 11 CRLs, demonstrating satisfactory prognostic prediction performance in BC 98. Besides, this model was also related to the assessment of immune checkpoint inhibitors (ICIs), TMB, m6A, and agent sensitization. Guo *et al.* reported a cuproptosis-associated risk model with 9 CRLs as an effective tool for independent prognostic prediction of BC 99. Moreover, the model also possessed immuno-predictive efficacy, and the low-risk group was able to respond better to immunotherapy, showing higher CD8+ T-cell infiltration and activation, and more TMB. Jiang *et al.* reported a novel signature based on 11 CRLs, and this signature could independently predict the prognosis and TMB 100. Furthermore, the high score cataloged by this signature showed high drug sensitivity for anti-CD276 therapy and imatinib, lapatinib, and pazopanib. Pan *et al.* also mined a model constructed from 10 CRLs and had similar predictive functions as independent prognostic predictors for BC 101. Zhang *et al.* adopted cuproptosis-related ferroptosis-related genes to investigate their expression patterns in predicting OS of BC. This study posed the capability of the pattern, and its relationship with steroid biosynthesis, ABC transporters, and drug sensitivity of AKT inhibitor VIII and cisplatin 102.

There is probably a robust link between cuproptosis and tumour immunity. Wang *et al.* explored the intricate relationship between cuproptosis and tumor immunity in TNBC to establish prognostic models based on miRNA and mRNA 103. By comparing the expression levels of CRGs between normal individuals and the TCGA-TNBC cohort, they identified 5 prognostic miRNAs (miR-203a-3p, miR-1277-3p, miR-135b-5p, miR-200c-3p, and miR-592) and 3 biomarkers (DENND5B, IGF1R, and MEF2C) that were significantly associated with TNBC prognosis. These factors might influence TNBC progression by affecting adipogenesis, inflammatory response, hormone metabolism, and the immune microenvironment. The cancer immunity cycle (CIC) is a crucial component of the cancer immune microenvironment, and might interact with cuproptosis to exert biological functions. Liu *et al.* utilized the TIP database and machine learning to identify 4 key cuproptosis-CIC interactions in BC and constructed a prognostic prediction model 104. Further experiments confirmed that HSPA9 was a critical protein that restricted BC growth and migration. These studies provide insights into the complex relationship between the cuproptosis-CIC network and the BC immune microenvironment **(Table 1)**.

## Cuproptosis-targeted therapy in BC

Evidence suggests that tumor cells undergoing regulatory death can remodel TME immunogenicity and intensity of immune effects, demonstrating the potential to inhibit cancer metastasis and recurrence 105. Other stromal and immune-type cells and components in the TME also undergo a definite degree of PCD effects, all of which exert a certain positive regulatory effect on tumor immunity 106,107. Currently, therapeutic modalities utilizing cuproptosis-targeted induced copper ion carriers in combination with small molecule drugs have demonstrated some tumor therapeutic potential 108.

Some TNBC-related CRGs, including ATP7A, PIK3CA, LIAS, and LIPT, were closely associated with some mutations and immune infiltration 71. Moreover, several drugs targeting cuproptosis were screened for the treatment of TNBC, including dasatinib in combination with ABT-737, Erastin or methotrexate, docetaxel/isprinib combination. Zinc pyrithione (ZnPT), an antifungal drug for seborrheic dermatitis, contributes to DNA damage and PARP-dependent energetic crisis 109. ZnPT is capable of exerting certain anticancer effects, including inhibition of PDAC tumor progression through suppression of SDCBP 110. Yang *et al.* demonstrated that ZnPT could induce TNBC cell death by disrupting the homeostasis of copper metabolism as well as triggering DLAT aggregation 111. Moreover, ZnPT inhibited TNBC cell proliferation, motility, stemness, and sensitized TNBC to chemotherapeutic effects, and ultimately inhibited TNBC development by inhibiting EGFR-PI3K-AKT and activating the MAPK signaling pathway. Deng *et al.* also constructed a risk model based on 4 CRLs, including C9orf163, PHC2-AS1, AC087741.1, and AL109824.1 after multiple analyses, and utilized this model to innovatively propose that the Hsp90 inhibitor 17-AAG was a candidate with potential therapeutic effects in high-risk groups 64.

Nano-complex-based drug-carrying systems can fully utilize the advantages of targeting, multiple synergistic functions, and safety 112-114. The induction and enhancement of PCD through nanocomplexes, such as cuproptosis, can not only promote tumor cell death, but also promote the release of immunogenic substances, promote immune activation, and amplify the therapeutic effect of the tumor 115. Of late, researchers focus on combining this advanced delivery method with cuproptosis induction, and have developed a series of novel drugs for BC. Inducing or enhancing cuproptosis by introducing high concentrations of copper and other agents is a common form. For instance, Chang *et al.* designed a polydopamine nanostructure (PDA-DTC/Cu) loaded with high concentrations of copper ions, which could enhance copper uptake and inhibit the expression of ATP7A and ATP7B, thereby reducing copper export and increasing intracellular copper accumulation to promote cuproptosis 116.

Additionally, tumor-associated macrophages were also stimulated to repolarize, alleviating immunosuppression within the tumor microenvironment. Overall, PDA-DTC/Cu demonstrated excellent tumor inhibitory effects by inducing cuproptosis as the core mechanism. Du *et al.* developed a self-reinforced bimetallic Mito-Jammer by constructing a hyaluronic acid -modified metal-organic framework loaded with doxorubicin and calcium peroxide 117. In 4T1 cell and 4T1 tumor-bearing models, the dissociation of calcium peroxide into hydrogen peroxide and Ca2+ enhanced the Cu2+-mediated Fenton reaction. This led to increased ROS production and mitochondrial damage, thereby significantly enhancing the sensitivity of tumor cells to cuproptosis and inhibiting tumor metastasis. In another study, Xu *et al.* synthesized disulfonamide-dimethylpyrimidine-phenanthroline-metal complexe HA-Cu, which demonstrated superior inhibitory effects on TNBC both *in vitro* and *in vivo* 118. HA-Cu effectively suppressed tumor survival and progression through a synergistic mechanism involving antiproliferative, antiangiogenic, anti-inflammatory, pro-apoptotic, and cuproptosis-inducing effects. HA-Cu inhibited the VEGF/VEGFR2 signaling pathway and enhanced cuproptosis in MDA-MB-231 cells by downregulating FDX1 expression and upregulating HSP70 expression. He *et al.* fabricated a type of Cu-chelated cyanine dye to deliver copper ions in different oxidation states to 4T1 cells and 4T1 tumor-bearing mice to investigate their roles in combating TNBC 119. They found that Cu+, in comparison to Cu2+, exhibited improved antitumor performance by generating more hydroxyl radicals through a faster pathway that did not require glutathione reduction. Zhang *et al.* developed a BPTES-loaded biomimetic Cu-doped polypyrrole nanoparticle (CuP) nanosystem (PCB), utilising various components to actively target tumor sites, reduce GSH levels, and induce oxidative stress and cuproptosis 120. PCB effectively inhibited the growth of both primary and distal tumors. By introducing glucose oxidase (GOx) and Cu sulfate, Lee *et al.* constructed a HD/BER/GOx/Cu hydrogel system for converting accumulating glucose into hydroxyl radicals and starvation/chemodynamic therapy by introducing glucose oxidase (GOx) and copper sulfate 121. The system enabled a multitude of tumor-killing effects through sustained release of drugs, including, notably, cupping-induced chemotherapy. In another study, Ning *et al.* designed a nanodelivery platform based on multifaceted induction of cupping, consisting of platelet vesicle (PV)-coated cuprous oxide nanoparticles (Cu2O)/TBP-2 cupping sensitization system (PTC) 122. This PTC system was able to release copper ions intracellularly, inhibit copper efflux, and further lead to lipoyl protein aggregation and iron-sulfur protein depletion, resulting in proteotoxic stress and cuproptosis. In this mechanism, PTC ultimately inhibited BC lung metastasis and enhanced T-cell immune effects.

**Figure 3:**
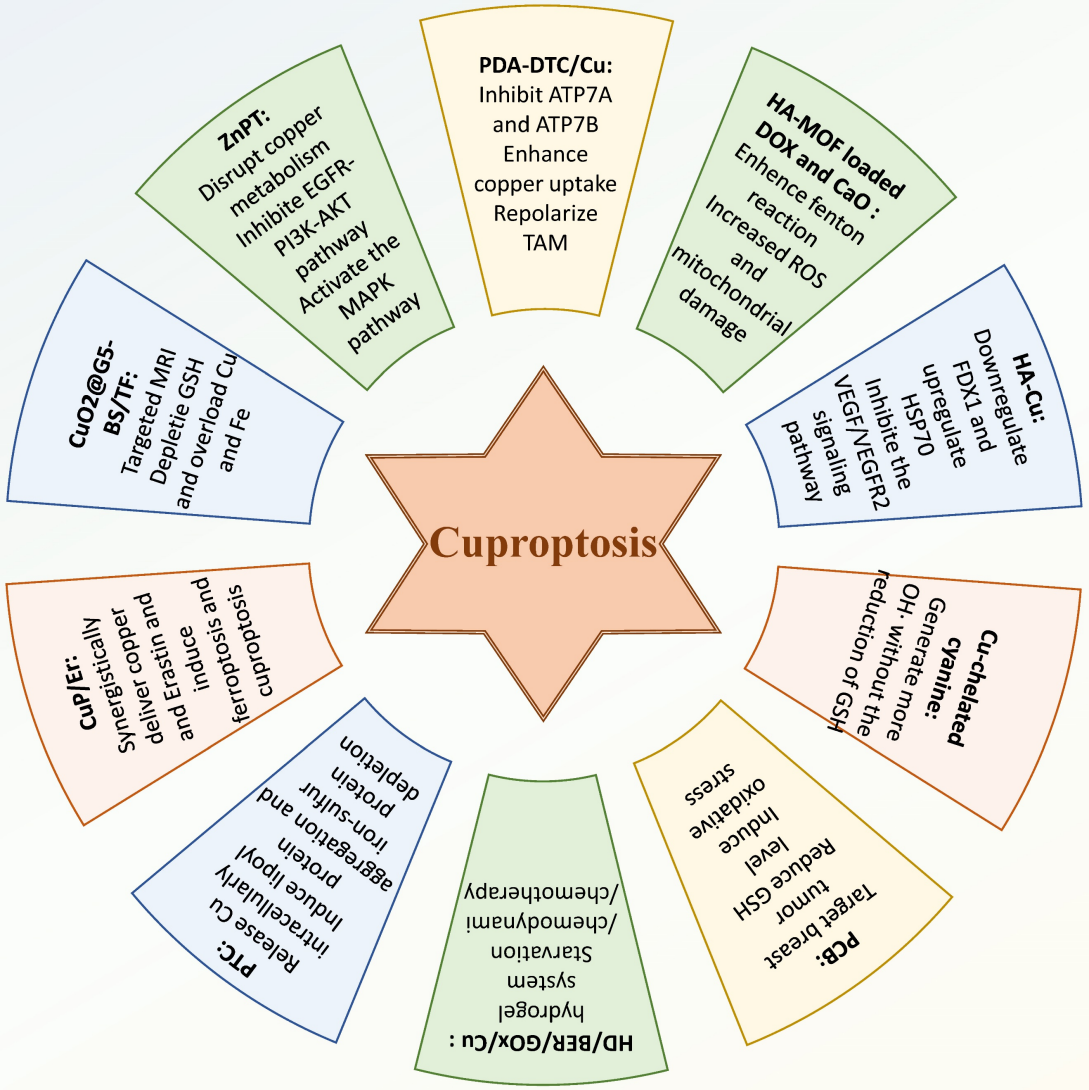
** Therapy targeting cuproptosis in BC.** Researchers have developed some drugs that can inhibit the development of BC by modulating cuproptosis in BC cells. These drugs, mostly in nano form, can induce cuproptosis in BC cells in a variety of ways or combine the induction of cuproptosis with other therapeutic approaches, yielding promising results in the treatment of BC.

Some studies have attempted to combine inducing cuproptosis with other antitumor therapies to enhance therapeutic efficacy. For example, Li *et al.* designed a core-shell nanoparticle, CuP/Er, which was capable of co-delivering copper and Erastin to cancer cells, thereby synergistically inducing ferroptosis and cuproptosis 123. Furthermore, CuP/Er was found to promote T cell proliferation and infiltration, enhancing the efficacy of immune checkpoint blockade therapy, resulted in inhibited survival and metastasis of murine colorectal adenocarcinoma and TNBC. Huang *et al.* developed a poly (amidoamine) dendrimer modified with p-carboxybenzenesulfonamide, loaded with copper peroxide nanoparticles, and combined with iron (Fe)-tannic acid (TF) networks to create a nanocomposite (CuO2@G5-BS/TF) 124. This nanocomposite effectively targeted and was internalized by 4T1 cells for targeted MRI. Furthermore, CuO2@G5-BS/TF induced ferroptosis and cuproptosis in 4T1 cells by depleting GSH and overloading copper and iron, while also alleviating the acidity of the tumor microenvironment, thereby inhibiting tumor metastasis. This modality offerd an attractive new method for targeted MRI and treatment of TNBC **(Figure 3)**.

## The re-thinking of cuproptosis in BC

Researchers have long noted the association between copper and cancer 125,126. As an essential transition metal in the body, the dysregulation of copper metabolism can lead to a range of cellular metabolic dysfunctions. The normal function of copper ions in cancer cells depends on the interactions between various copper-related proteins. These include proteins involved in copper transmembrane transport such as CTR1, SLC25A3, ATP7A/B, proteins responsible for binding and storing copper ions like MT and glutathione, and copper chaperone proteins such as ATOX1, CCS, and COX17. The interaction of these proteins is fundamental in maintaining intracellular copper homeostasis 127-130. By engaging key molecules in these pathways, intracellular copper can directly participate in regulating multiple signaling pathways in tumor cells, including receptor tyrosine kinase (RTK)-related signaling pathways, phosphoinositide 3-kinase (PI3K)-AKT signaling pathway, mitogen-activated protein kinase (MAPK) signaling pathway, and Notch pathway 131-134. These pathways influence tumor metabolism, proliferation, angiogenesis, and metastasis.

Elevated copper levels have been found in the tumors and serum of various animal models and cancer patients, including prostate cancer, BC, thyroid cancer, lung cancer, and gastric cancer. In lung cancer patients, higher serum copper ion concentrations are associated with worse clinical staging in northeast china 135. Based on the major discovery that copper can induce cell death, cuproptosis is considered a promising direction for cancer therapy. By regulating copper levels in tumour cells, normal biochemical processes can be disrupted, thereby inhibiting the proliferation and survival of cancer cells. Consequently, the profound interconnection between cuproptosis and cancer represents a burgeoning area of interest. Tsvetkov *et al.* identified 10 genes significantly associated with the risk of cuproptosis through genome-wide knockout screening. Further analysis revealed that SLC31A1, ATP7A, and ATP7B were deeply involved in regulating intracellular copper homeostasis. These 13 genes were considered critical cuproptosis genes (CKGs) 28. Researchers have focused on the expression levels and clinical significance of these CKGs and some CRGs in different tumors. Interestingly, CKGs and CRGs seem to play different roles in various tumors. For instance, FDX1 encodes Ferredoxin1, which participates in multiple redox reactions and enhances the toxicity of copper ions by reducing them, playing a central role in cuproptosis 136,137. Elevated levels of FDX1 are observed in glioblastoma and female reproductive tumors, whereas its expression is downregulated in solid tumors like lung adenocarcinoma and hepatocellular carcinoma 138. High levels of FDX1 are associated with poor prognosis in patients with head and neck squamous cell carcinoma and low-grade glioma, but predict better prognosis in patients with cervical squamous cell carcinoma and clear cell renal cell carcinoma. Similar effects can be observed in another CKG, namely LIAS. The high expression of LIAS is associated with poor prognosis in lung cancer, whereas in KIRC and ovarian cancer, high expression of LIAS indicates better prognosis 139.

Given that research on cuproptosis is still in its early stages, further studies are needed to elucidate the reasons for these differential expressions in various tumors and the specific mechanisms by which these differences impact prognosis. On one hand, this may be because these CKGs are involved not only in cuproptosis-related pathways but also in regulating biological processes, such as immune infiltration, energy metabolism, and methylation. In pan-cancer analyses, these genes exhibit different characteristics in various tumors 140. On the other hand, it may also be related to the inherent characteristics of some tumors. For example, there is a significant correlation between cuproptosis and mitochondrial metabolic levels, and certain tumors inherently exhibit higher levels of mitochondrial metabolism, such as melanoma, BC, and leukemia 141,142. Therefore, inducing cuproptosis as a means to treat tumors is more likely to yield ideal therapeutic effects in these types of cancers.

BC exhibits significant heterogeneity, both among different patients and within tumors from the same individual, primarily characterized by variations in hormone receptor status, HER2 expression, mutational landscape, and intratumoral heterogeneity 143,144. Hormone receptor-positive (ER+ and/or PR+) and TNBC differ markedly in their biological characteristics, treatment responses, and prognoses 145,146. HER2-positive tumors are particularly responsive to HER2-targeted therapies like trastuzumab but may exhibit reduced sensitivity to other treatments 147. Common genetic mutations in BC include TP53, PIK3CA, and GATA3, which influence the malignancy and therapeutic responses of the tumors 148,149. Additionally, intra-tumor heterogeneity is evident, with different cellular subpopulations within a single breast tumor displaying variations in cell morphology, proliferative capacity, and gene expression patterns 150. This extensive molecular heterogeneity not only dictates the biological behaviors of BC but also directly impacts its response to various therapeutic strategies.

BC cells exhibit a unique copper metabolism, requiring higher amounts of copper, which makes them more sensitive to cuproptosis inducers. Research indicates that dysregulation in copper metabolism is closely related to malignant progression of BC. High expression of copper transport proteins, such as SLC31A1, facilitates copper uptake, leading to the occurrence of cuproptosis 151. Dysregulation of copper metabolism-related genes affects copper homeostasis, causing intracellular copper ion accumulation and triggering cuproptosis. By regulating the expression of these genes, the growth and proliferation of BC cells can be effectively controlled. For instance, TNBC shows a high copper demand and sensitivity to cuproptosis inducers like ZnPT 111. This disrupts copper metabolic homeostasis and induces cell death by DLAT aggregation, thereby inhibiting proliferation, migration, and stemness. The unique relationship between cuproptosis and BC highlights the role of molecular heterogeneity in determining therapeutic responses. Different BC subtypes exhibit distinct responses to cuproptosis-inducing therapies 152. Hormone receptor-positive BC cells are sensitive to hormone therapy but may respond weakly to cuproptosis inducers; however, this can be improved through combination treatments. HER2-positive BC cells, while responsive to HER2-targeted therapies like trastuzumab, require further investigation to understand their response to cuproptosis inducers. Combining HER2-targeted therapies with cuproptosis inducers could be an effective strategy, as copper metabolism may interact with the HER2 signaling pathway, influencing cell growth and proliferation. TNBC, with its high dependency on copper metabolism, presents significant potential for the application of cuproptosis inducers, offering a promising approach for its treatment.

## Discussion

In this study, we systematically elucidated the cuproptosis mechanism, the regulatory mechanism of cuproptosis on BC, the application of cuproptosis in the diagnosis and prediction of BC, and the therapeutic strategy of BC based on cuproptosis. However, there are some limitations and outlooks that need close attention in the current field.

First, cuproptosis has been poorly studied in BC in general, and most of these studies are still in the data mining related to cuproptosis. The biological processes of BC involve many different types of PCD modalities, including apoptosis, ferroptosis, autophagy, and cuproptosis 153,154. Compared to other forms of PCD, experiments related to cell, animal, and drug mining for cuproptosis are less reported. This implies that the roles and mechanisms of cuproptosis-related molecules in BC that have been mined so far are still full of mysteries. Different PCD patterns are co-existed in the BC progression, as evidenced by that simultaneous inductions of cuproptosis and ferroptosis in BC cells inhibit BC cell malignant behaviors 124. Furthermore, BC is a highly heterogeneous solid tissue with a complex composition, which also encompasses complex pathological and molecular typing. The responsiveness of different BC subtypes to cuproptosis is significantly different. Previous studies of cuproptosis often did not give a clear qualification of the subtypes in BC, and only in a few studies did they indicate the subtypes as TNBC or ER+ BC. The possibility that different subtypes may differ in their responsiveness to cuproptosis is a subsequent point that needs to be addressed urgently. It is also important to note that most of the cuproptosis focuses on the tumor cells themselves in the TME, while the cuproptosis effects of other stromal and immune cells have been reported. Overall, cuproptosis, as an emerging research hotspot, still lacks a large number of high-quality basic studies to confirm its role and mechanism in BC.

Cuproptosis has shown significant potential in the diagnosis of BC. In BC diagnostics, CRGs and CRLs provided valuable key information, such as rich tumor immunoprediction and clinical prediction. Risk models constructed from different CRGs have been widely recognized. BC is a heterogeneous entity composed of a variety of immune cells, stromal cells, and tumor cells 155,156. CRG-related model is not only capable of determining the prognosis, but also analyzing the characteristics of the cellular components of the entity microenvironment, the intensity of immunity, the TMB, and the assessment of the responsiveness to treatment. These provide rational therapeutic windows and strategies for precise therapies of BC. Risk models constructed based on multiple CRGs or combining CRGs and other ncRNAs together demonstrated excellent predictive capabilities. However, cuproptosis-based markers are still in the very early stages of validation. Nost of the studies are still at the stage of basic research and data mining, lacking large-scale clinical validation. In addition, the clinical translation of CRG models faces challenges, including the need for standardized detection methods and large-scale clinical trials. More in-depth retrospective and prospective studies are necessary to establish cuproptosis as a useful adjunct to traditional diagnostic methods in clinical practice.

Finally, in tumor therapy, the antitumor potential and safety of a large number of copper chelators and copper complexes have been preliminarily demonstrated in preclinical cell and animal studies. Copper-related drugs represented by DSF and TTM can produce synergistic therapeutic effects with other chemotherapeutic agents, inhibiting tumor resistance, suppressing tumor recurrence as well as sensitizing drug effects 157,158. However, compared with copper homeostasis-related genetic diseases, the application of cuproptosis-targeted therapy for tumors is not well established. In BC, the relevant therapeutic applications are in the cellular and animal studies of cuproptosis induction, and the specific mechanisms and applicable individuals have not been fully analyzed. Moreover, the number of these studies is relatively small, and continued exploration is necessary for the treatment of BC. In addition, since curpoptosis is a novel PCD mechanism that has been relatively little and superficially studied in BC, the modulation of tumor immunity has not been given a clear exploration. However, PCD produces damage-associated molecular patterns that contribute to antitumor immunotherapy, stimulate inflammatory responses, and mediate the activation of a variety of immune cells and factors, making the induction of PCD a potential immunotherapeutic strategy 159,160. Moreover. These types of cell death are not independent, but are closely cascaded or coordinated, and the use of nanomedicines can induce a combination of PCDs for treatment.

Finally, the increased copper requirement of tumour cells may be related to the role of copper in cell proliferation and metabolic activities. However, excess copper confers an anti-tumour effect by inducing copper toxicity, which is toxic to cancer cells. Copper is also an ion essential for normal physiological cellular activity, and the concentration must be maintained within a specific physiological range. To achieve therapeutic copper overload while minimizing damage to normal tissues, precise control over copper delivery and concentration is crucial. Additionally, targeted delivery systems or selective activation mechanisms could help specifically induce cuproptosis within the tumor microenvironment, thereby reducing adverse effects on normal cells.

## Conclusion

Collectively, the delicate equilibrium of copper ions within the human body is essential for maintaining cellular homeostasis and is intricately linked to the pathogenesis and progression of BC. Cuproptosis has gradually emerged to play an increasingly prominent role in BC, including influencing the proliferation, invasion, prognosis, and treatment of BC. CRGs and CRLs based on cuproptosis have also demonstrated rich predictive information for BC. In addition, therapeutic strategies targeting cuproptosis are capable of capabilities to elicit positive therapeutic effects. Compared with other PCD types, cuproptosis currently has a long way to go in BC, which will provide a theoretical basis for cuproptosis-targeted therapy. Lasly, cuproptosis sheds light on the copper dependency and metabolic characteristics of BC, providing new avenues for precision medicine and personalized therapeutic strategies.

## Figures and Tables

**Table 1:** Cuproptosis-related applications in Breast Cancer diagnosis.

Related Molecule	Diagnosis application	Reference
SLC31A1	Integrated SLC31A1 with other clinical parameters, such as age, T-stage, N-stage, and clinical staging.	Ref.17
PDHA1	PDHA1 was a regulator of BC malignant progression and its expression was tightly correlated with the infiltration of a variety of immune cells.	Ref.81
DLAT, SLC31A2, SLC25A3, ATOX1	Classifying HER2+ BC patients into low-risk and high-risk groups by risk scores, effectively assess the prognosis of HER2+ BC patients.	Ref.53
CASC 8, LINC 02188, LINC 00511, GRIK 1-AS 1, ROCR	The AUC of the model constructed for the prediction of breast cancer subtypes was 0.816 and the F1 score was 0.57. In addition, the precision of the model was 0.56, the specificity was 0.81, and the sensitivity (Sn) was 0.61.	Ref.84
PTPRN2, SCARB1, SLC37A2, YES1, LY6D, and NOTCH3	With a high AUC score over 0.85 in TCGA dataset, high- and low-risk groups distinguished by the model had distinct prognosis and immune infiltration.	Ref.85
TFF1, GREB1, SCUBE2, MMP7, SUSD3, CHI3L1, STC2, SLC7A5, MAPT, FABP7 and CHAD	Significant differences existed between the high- and low-risk groups in terms of OS, immune status, and sensitivity to chemotherapy.	Ref.86
DKN2A、MTF1、PDHA1、DLD、LIPT1 and FDX1	The model predicted the OS rate with an accuracy that ranged from medium to high.	Ref.62
FDX1, LIAS, LIPT1, DLD, PDHB, ATP7B and CDKN2A	The model specified a higher risk of recurrence with a high CRG score.	Ref.89
PPIC-AS1, MME-AS1, AC012676.3, MFF-DT, OTUD6B-AS1, AL109936.9, AL807757.2, AL118556.1, AL137847.1	The predictive model classified patients into high-risk and low-risk groups, with a significantly higher mortality rate in the high-risk group. The results of the C-index curves indicated that the predictive model possesses a higher degree of accuracy in assessing prognosis than the clinicopathological features.	Ref.93
AC104211.1, AL162595.1, LINC02166, AC007996.1, AC005865.2, AC007493.1, AL449423.1, AC004847.1, AL161910.1, LINC01863, AC018978.1, AC092384.3, AL356740.2	The model grouped patients according to thier scores, and the AUC values of the risk scores for predicting 1-, 3-, and 5-year survival of BC in the validation set were 0.73, 0.68, and 0.71, respectively.	Ref.94
USP2-AS1, NIFK-AS1	BCCuS-high group and BCCuS-low group showed significant differences in gene mutation frequency, immune function, TIDE (tumor immune dysfunction and exclusion) score and other phenotypes.	Ref.95
C2orf91(LINC02898), PRKAR1B-AS1, AC012213.3, AL137847.1, MFF-DT	These CRLs were closely related to immune responsiveness, the OS of patients in the high-risk group was lower than that in the low-risk group.	Ref.96
AL139241.1、MFF-DT、AL451123.1、AC009120.5、AL137847.1、HECW 2-AS 1、LINC 01031、NIFK-AS 1、AL592301.1、U73166.1	The model categorized patients into high- and low-risk groups, the risk score was significantly relevant to the survival of BRCA patients and demonstrated a better predictive ability for 1- and 3-year survival with the AUC of approximately 0.74, as well as the potential of predicting immunotherapy responsiveness	Ref.97
AL023882.1, AC091588.1, AC138028.2, AC027514.1, AL592301.1, LRRC8C-DT, MFF-DT, NIFK-AS1, MECOM-AS1, OTUD6B-AS1 and RNF32-AS1	Patients in the high-risk group distinguished by this model face heavier tumor mutational load (TMB), have suppressed anti-tumor immunity, and possess a worse prognosis.	Ref.98
LRRC8C-DT, TDRKH-AS1, SAMMSON, SIAH2-AS1, WDFY3-AS2, LINC00393, ARHGAP28-AS1, PCAT18, and LINC01711	Patients with BC in the low-risk groups showed better clinical outcomes different immune cell infiltrations, with better response to immunotherapies.	Ref.99
GORAB-AS1, AC 079922.2, AL 589765.4, AC 005696.4, Cytor, ZNF 197-AS1, AC 002398.1, AL 451085.3, YTH DF 3-AS1, AC 008771.1, LINC 02446	The model showed the ability to independently predict the prognosis and TMB, with the AUC values for ROC of 1-, 3-, and 5-year risk were 0.849, 0.779, and 0.794, respectively.	Ref.100
AL118556.1, AL451123.1, MFF-DT, AL133243.2, ZKSCAN7-AS1, AC012676.3, AC009506.1, AC079766.1, MIR1915HG, AC138028.2	ROC and PCA showed that the model has accurate prediction ability, with higher tumor mutation burden and high TMB-resulted lower survival in high-risk group.	Ref.101
ANO6, CHAC1, CHMP6, CS, EMC2, G6PD, GPX4, PANX1, PIK3CA, SLC7A5, and SOCS1	The model demonstrated differences in steroid biosynthesis, ABC transporters, and drug sensitivity to AKT inhibitor VIII and cisplatin between subgroups.	Ref.102
miR-203a-3p, miR-1277-3p, miR-135b-5p, miR-200c-3p, miR-592, DENND5B, IGF1R, MEF2C	Patients were categorised into high- and low-risk groups based on risk scores from the model, which combined the cancer status and pathological staging to predict 1/3/5-year patient survival and provide new therapeutic targets for BC.	Ref.103
CXCL13, HSPA2, HSPA9, MICB	A nomogram model was developed to predict survival in BC patients by linking risk scores to clinicopathological features and revealed that targeting HSPA9 could be a potential means of improving breast cancer outcomes.	Ref.104

## Abbreviations

ATOX1: copper molecular chaperone antioxidant 1
ATP7A: ATPase copper transporting alpha
ATP7B: ATPase copper transporting beta
BC: breast cancer
CAFs: cancer-associated fibroblasts
CCO: cytochrome oxidase C
CDI: cell death index
CDKN2A: cyclin-dependent kinase inhibitor 2A
CIC: cancer immunity cycle
CKGs: critical cuproptosis genes
CRGs: cuproptosis-related genes
CRLs: cuproptosis-related lncRNAs
CTR1: copper transport protein 1
DLAT: dihydrolipoamide S-acetyltransferase
DLD: dihydrolipoyl dehydrogenase
DMFS: distant metastasis-free survival
ER+: estrogen receptor-positive
GOx: glucose oxidase
ICIs: immune checkpoint inhibitors
MAPK: mitogen-activated protein kinase
OS: overall survival
PV: platelet vesicle
PCDs: programmed cell deaths
PDHA1: pyruvate dehydrogenase E1 component subunit alpha
PI3K: phosphoinositide 3-kinase
ROS: reactive oxygen species
RFS: recurrence-free survival
RTK: receptor tyrosine kinase
SCFA: short-chain fatty acid
STEAP: six-transmembrane epithelial antigen of the prostate
SLC31A1: solute carrier family 31 member 1
SOD1: superoxide dismutase 1
TCA: tricarboxylic acid
TLR4: Toll-like receptor 4
TME: tumor microenvironment
TMB: tumor mutation burden
TNBC: triple negative breast cancer
ZnPT: zinc pyrithione
